# A Fiber Optic Sensor Using a Molecularly Imprinted Chitosan Membrane Coating on a Fiber Surface as a Transducer for Discriminating 4-Nitrophenol from Its Positional Isomers

**DOI:** 10.3390/s26020398

**Published:** 2026-01-08

**Authors:** Myra Arana, Shiquan Tao

**Affiliations:** Department of Chemistry and Physics, West Texas A&M University, WTAMU Box 60732, Canyon, TX 79016, USA

**Keywords:** optical fiber chemical sensor, molecularly imprinted polymer, 4-nitrophenol, solid phase microextraction, bent optical fiber probe

## Abstract

An optical fiber chemical sensor using a molecularly imprinted chitosan membrane coated on the surface of a bent optical fiber probe was developed for selectively analyzing 4-nitrophenol (4-NP) in water samples. When the sensor probe is exposed to a water sample, the chitosan MIP membrane extracts/concentrates 4-NP from the water sample into the membrane. The 4-NP extracted into the membrane was detected by passing a light beam through the optical fiber and the interaction of the 4-NP in the membrane with an evanescent wave of light guided through the optical fiber was detected as a sensing signal. This sensor detects the intrinsic optical absorption signal of 4-NP itself as a sensing signal. No chemical reagent was needed in analyzing this compound in a sample. The sensor is reversible, can be used for continuous monitoring of 4-NP in a sample, and has a quick response with a response time of 5 min. The sensor has high sensitivity and selectivity because the MIP membrane selectively concentrates 4-NP by 1.4 × 10^4^ times into the membrane from a sample solution, but blocks out interference species, including its isomers and derivatives, from entering the membrane. The sensor achieved a detection limit of 2.5 ng/mL (0.018 µM), which is lower than most reported analytical techniques for analyzing this compound in water samples. This sensor can discriminate 4-NP from its isomers and derivatives, such as 2-NP, 3-NP, 2-Cl-4-NP, and 2,4-di-NP, with a selectivity factor ranging from 104 to 1922. This is the first reported case of an MIP-based optical fiber chemical sensor with the capability of discriminating an organic compound from its closely related positional isomers, which demonstrates the high selectivity nature of the MIP-based optical fiber chemical sensor technique. The sensor has been used for analyzing 4-NP in a standard addition sample. The obtained recovery rate ranged from 93% to 101%, demonstrating the application potential of this sensor in water quality analysis.

## 1. Introduction

4-nitrophenol (4-NP) is a powerful carcinogen which is in the US Environmental Protection Agency’s priority toxic pollutant list [1,2]. Sources of 4-NP contamination include leather processing, the synthesis of many industrial products such as explosives, herbicides, pesticides, petrochemicals, pharmaceuticals, and synthetic dyes [3,4]. In addition, the degradation of pesticides such as parathion and nitrofen also generates 4-NP in the environment [5,6,7]. It is difficult to remove this compound once it is entered into the environment, such as in ground/underground water, due to its high solubility in water and its stability in the environment. Remediation methods to remove this compound include electrochemical reduction, adsorption into biochars, microwave irradiation, chemical reduction, and bioremediation [8,9,10]. Analytical technologies capable of quick analyzing or continuous monitoring of 4-NP concentration in environmental samples are needed for cost-effective removal of this pollutant.

4-NP is an oxidant and it can be reduced on an electrode with appropriately applied potential. Therefore, electrochemical analytical techniques have been developed for analyzing this compound in environmental samples. Electrochemical detection techniques such as cyclic voltammetry, linear sweeping voltammetry, and differential pulse voltammetry have been reported with different electrodes for 4-NP analysis [8,11,12,13]. However, redox reaction of other compounds co-existing with 4-NP in sample solutions causes interference to most of these electrochemical methods. Intensive efforts have been devoted to modifying electrodes with organic polymers, noble metal nanomaterials, carbon nanotube (single wall or multiwall), and reduced graphene oxide in order to increase the selectivity, but with only limited success [13].

4-NP absorbs light, with a peak absorption wavelength of 320 nm [14,15]. However, it is almost impossible to use this intrinsic optical absorption property to directly analyze this compound in environmental samples due to the co-existence of interference species. A separation technique must be applied to separate this compound from the sample matrix before detection with optical absorption spectrometry. Solvent extraction, solid-phase microextraction (SPME), HPLC, and capillary electrophoresis have been reported for separating 4-NP from environmental sample, followed by UV/Vis absorption spectrometric detection [14,15,16,17]. There are also other analysis techniques, such as fluorescence spectrometry and mass spectrometry, which having been reported for detecting 4-NP after separating the analyte from sample matrix [18,19,20]. These traditional analytical techniques involve sophisticated instruments (HPLC or mass spectrometers) which have to be installed in a well-controlled laboratory environment and operated by well-trained scientists or using tedious procedures (such as extraction separation and adsorption/desorption). More convenient, quick, and field-deployable techniques are desired for monitoring environmental remediation processes of removing this pollutant.

Molecularly imprinted polymer (MIP) is a recently developed material used for separation in environmental remediation as well as in analytical chemistry [21,22]. An MIP is synthesized by cross-linking a monomer in the existence of a template molecule. The template compound is enclosed in the formed polymer after polymerization with specific bonding (covalent bond, hydrogen bond, or ionic attraction) with the polymer’s side chain. The template molecules can be washed out of the formed polymer with an appropriate reagent. After removing the template, a polymer with pores which are geometrically complementary to the shape of the template molecule, with a specific bonding site to bond the template molecule, was formed. Therefore, an MIP made from such a process will be highly selective in extracting the template molecules from a sample solution. This material can be used for separating an analyte which has been used as a template for synthesizing the MIP from the sample matrix, before applying an appropriate technique to detect the compound.

**Figure 1:**
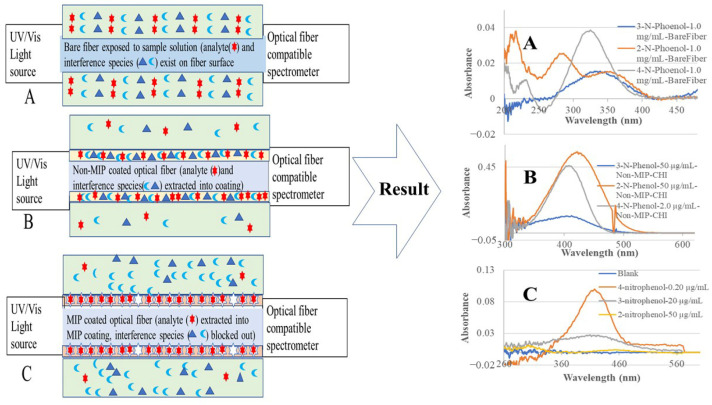
The diagrammatic graphs (**left**) show the difference in exposing a bare fiber (A), a non-MIP-membrane-coated fiber (B) and an MIP-membrane coated fiber (C) to a sample solution containing an analyte and an interference species. The non-MIP membrane extracts/concentrates both analyte and interference species. The MIP membrane selectively extracts/concentrates the analyte, but blocks out the interference species. The absorption spectra on the (**right**) side demonstrate the sensitivity and selectivity improvement that resulted from non-MIP/MIP membrane extraction.

In recent developments, we integrated MIP-SPME with our previously developed evanescent wave optical fiber chemical sensor (EW-OFCS) technique using a bent optical fiber probe (BOFP) as an optical waveguide [23,24,25,26,27]. The advantages of this MIP-SPME-EW-OFCS technique are diagrammatically demonstrated in Figure 1. MIP-SPME selectively concentrates an analyte from a sample solution and at the same time blocks out interference species from entering the membrane. Although optical fiber EW absorption spectrometry is a technique with limited sensitivity due to limited interaction pass-length [28,29], our MIP-SPME-EW-OFCSs achieved high sensitivity with detection limits in a sub-µg/mL range, due to MIP-SPME’s high concentration factor. Compared with a non-MIP membrane, a MIP membrane is highly selective in extracting target analyte due to MIP’s selective adsorption nature. Our reported MIP-SPME-EW-OFCSs have been successfully used to analyze trace organic acids and caffeine in complex food and pharmaceutical products [23,24,25,26,27].

In this work, we integrated the MIP-SPME with the EW-OFCS technique in an effort to develop a chemical sensor for quick analysis of 4-NP in environmental samples. A chitosan MIP membrane was synthesized with 4-NP as a template and was coated on the surface of a BOFP. After washing out the 4-NP template from the membrane, the BOFP can be used for analyzing 4-NP by simply inserting the bent probe into a water sample and monitoring 4-NP’s intrinsic optical absorption signal at 410 nm by passing a light beam through the BOFP.

Compared with traditional techniques such as HPLC-UV for analyzing 4-NP in water samples, this sensor has following advantages: 1. a much lower cost (a sensor can be constructed with a 410 nm light emitting diode, an MIP-membrane-coated BOFP, and a photodiode and read-out computer program with a total cost < USD 500); 2. a quick sample analysis time (<10 min for analyzing a sample); 3. the capability of real-time continuous monitoring of a remediation process; 4. portability for field applications.

## 2. Experimental

### 2.1. Chemicals

Deionized (DI) water was used for preparing aqueous solutions in this work. The 2-nitrophenol, 3-nitrophenol, and 4-nitrophenol were all purchased from Sigma-Aldrich (C_6_H_5_NO_3_, >98%, Sigma-Aldrich, St. Louis, MO, USA). These reagents were directly used for preparing standard NP solutions without further purification. In preparing an NP standard solution, a solid reagent powder was weighted with an analytical balance and directly dissolved in DI water of appropriate volume.

A chitosan solution of 10 mg/mL in 2% of acetic acid solution was prepared by mixing 0.50 g chitosan powder (chitosan from shrimp shells, practical grade, Sigma-Aldrich) with 49 mL DI water and 1.0 mL glacial acetic acid (CH_3_CO_2_H, ReagentPlus, >99%, Sigma-Aldrich), with magnetic stirring at room temperature for 12 h.

A 4-NP-templated chitosan MIP coating solution was prepared by mixing 1 mL of the chitosan solution, 0.10 mL of a 5 mg/mL 4-NP solution, and 50 µL of 25% glutaraldehyde (GTA, OHC(CH_2_)_3_CHO, grade II, 25% solution in water, Sigma-Aldrich) solution. The mixture was vigorously shaken for 1 min before coating. The coating solution was prepared immediately before coating the surface of a BOFP with the solution. 2-NP and 3-NP templated chitosan MIP coating solutions were also prepared using the same volumes of chitosan, GTA, and template solutions, but the concentrations of 2-NP and 3-NP were 1 mg/mL.

All other chemical reagents used in this work were analytical-reagent grade and were used without further purification.

### 2.2. Instruments

An optical fiber compatible UV/Vis spectrometer (USB4000, OceanOptics, Dunedin, FL, USA) was used together with computer software (OceanView, OceanOptics) for recording the fiber optic spectrum (the intensity, absorbance, time response at selected wavelength, etc.). An optical fiber-compatible deuterium/tungsten light source (DH2000, OceanOptics) was used as the light source for the fiber optic spectrometric measurement. A Nicolet iS5 FTIR spectrometer (Thermo Fisher Scientific, Madison, WI, USA) was used to study a pure 4-NP powder sample, membranes of chitosan, a GTA-crosslinked chitosan, and a 4-NP-templated chitosan MIP. This instrument comes with a diamond crystal attenuated total reflection (ATR) accessory. ATR-FTIR spectra of the 4-NP and polymer membranes were recorded by placing the powder sample or polymer membrane on the ATR sample adaptor and pressing the sample tightly to the diamond crystal and then recording the transmittance spectrum with air as a reference sample.

### 2.3. Preparation and Coating of the BOFP with GTA-Crosslinked Chitosan and 2-NP-, 3-NP-, and 4-NP-Templated Chitosan MIP Membranes

A deep-UV-transmittance-enhanced optical fiber (FG200AEA, fiber core/cladding diameter = 200/220 µm, Thorlabs, Newtown, NJ, USA) was used to prepare a “U”-shaped BOFP, because some of the compounds tested in this work absorb light below 350 nm. The center part of a 30 cm straight fiber was inserted into a small butane flame. About 1 cm of the fiber’s jacket and cladding (both are organic polymers) were burnt off in the butane flame. The optical fiber core was further forced to bend in the high-temperature flame to form a “U”-shaped structure. After cooling down to room temperature, the “U”-shaped optical fiber core was inserted into a K_2_Cr_2_O_7_/H_2_SO_4_ wash solution (caution: this wash solution is very corrosive and should be used with caution) to wash off any organic species from the optical fiber core’s surface. The “U”-shaped part of the BOFP was then inserted into a concentrated hydrofluoric acid solution (caution: concentrated hydrofluoric acid is poisonous and should be handled with caution) for 20 min in order to reduce the diameter of the “U”-shaped part of the fiber core to increase the EW-OFCS’s sensitivity [30,31]. Due to the limited availability of microscope, the diameter of the “U”-shaped part was not precisely measured in this work. The optical fiber probe was then rinsed with DI water and inserted into a 1 M NaOH solution for >20 min to activate the -OH groups on the optical fiber core’s surface. The BOFP was rinsed with water again and air dried before being coated with a chitosan membrane.

In coating a chitosan MIP membrane on the BOFP’s surface, the “U”-shaped probe was inserted into the corresponding chitosan MIP coating solution and removed slowly. The coated BOFP was allowed to gelate overnight in air before use.

A single BOFP was used in this work to prepare different chitosan membrane coatings (non-MIP chitosan, 2-NP-templated MIP, 3-NP-templated MIP, and 4-NP-templated MIP) on its surface. It is possible to compare the sensitivity of SPME-EW-OFCSs of different membranes using a single BOFP because the sensitivity of the BOFP-EW-OFCS depends on the BOFP’s structure (the “U”-shaped fiber core diameter and the bending diameter) [28,29]. In order to use a single BOFP to prepare different chitosan membrane coatings, a K_2_Cr_2_O_7_/H_2_SO_4_ wash solution was used to remove a non-MIP chitosan membrane or a chitosan MIP membrane from the BOFP surface by inserting the bent part of the BOFP into the wash solution for >12 h. This polymer-removed BOFP was then activated using the 1 M NaOH solution and rinsed with DI water before the next coating operation.

### 2.4. ATR-FTIR Studies of Membranes of Chitosan, GTA-Crosslinked Chitosan, 4-NP-Templated Chitosan, and a Pure 4-NP Powder Sample

ATR-FTIR was used to study pure 4-NP and the membranes of the polymers involved in this work, including chitosan, GTA-crosslinked chitosan, and a 4-NP-templated chitosan MIP, in order to verify 4-NP’s immobilization in the chitosan MIP. The 4-NP reagent sample was ground with a ceramic pestle and mortar before placing the reagent sample onto the iS5′s ATR adaptor to record its FTIR spectrum. The polymer membranes were made by placing the following polymer solutions onto glass microscopic slides and allowing the solution to gelate/dry for >24 h: 1 mL of the 10 mg/mL chitosan in a 2% acetic acid solution; a mixture of 1 mL of the 10 mg/mL chitosan solution plus 50 µL of the 25% GTA solution; and a mixture of 1 mL of the 10 mg/mL chitosan solution, 50 µL of the 25% GTA solution, and 100 µL of a 5 mg/mL 4-NP solution. The membranes formed were removed carefully from the glass slides with a sharp blade and placed onto the ATR adaptor to record their FTIR spectra.

### 2.5. Fiber Optic EW Spectrometry Monitoring of the Washing out of Template Molecules from the Chitosan MIP Membrane

The two ends of a chitosan-MIP-membrane-coated BOFP were connected to the USB 4000 optical fiber-compatible spectrometer and the DH2000 combo light source with SMA connectors, respectively. When the BOFP was inserted into water in a small reagent bottle, the light intensity spectrum was recorded as a reference intensity spectrum for measuring the fiber optic EW absorption spectrum. During the time the BOPF was inserted in DI water to wash out the template molecules from the membrane, the optical fiber EW absorption spectrum was monitored and recorded at different time intervals.

### 2.6. Analysis of 4-NP in Aqueous Sample Solutions with the Chitosan-MIP-Coated BOFP

After washing out the 4-NP template molecules from the chitosan membrane, a new reference light intensity spectrum was recorded for measuring the fiber optic EW absorption spectrum of a 4-NP sample solution. The “U”-shaped probe was then inserted into a 4-NP sample solution and the fiber optic EW absorption spectrum was recorded 6 min after the probe was inserted into the sample solution.

## 3. Results and Discussion

### 3.1. FTIR Spectra Indicting the Immobilization of 4-NP in the Chitosan MIP Membrane

**Figure 2:**
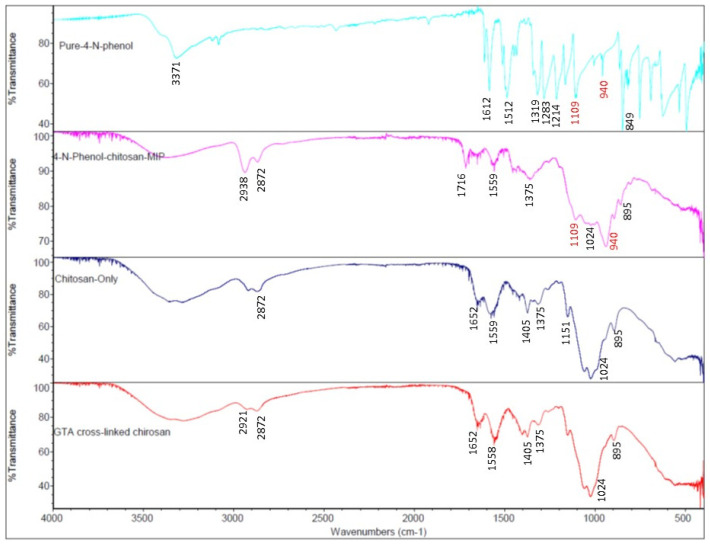
FTIR spectra of three polymer membranes: pure chitosan, GTA-crosslinked chitosan, a 4-NP-templated chitosan MIP, and a pure 4-NP sample. These FTIR spectra clearly indicate that 4-NP was immobilized in the 4-NP-templated MIP membrane.

FTIR is a useful optical spectrometric method for identifying chemical species. In this work, the FTIR spectra of three polymer membranes chitosan, GTA-crosslinked chitosan, a 4-NP-templated chitosan MIP, and pure 4-nitrophenol were recorded as shown in Figure 2. The peak at 1109 cm^−1^ in the FTIR spectra of the 4-NP-templated chitosan MIP membrane and the pure 4-NP powder sample originated from nitrophenol’s C-NO_2_ vibration. This peak was also observed in the FTIR spectra of a previously published work which used a chitosan MIP to remove 4-NP from a contaminated waterbody [32]. The peak at 940 cm^−1^ was from 4-NP’s aromatic C-H out-of-plane bending vibration. This peak was also reported in the published FTIR spectra of a 4-NP-templated chitosan MIP and a pure 4-NP sample [32,33]. These two FTIR peaks appeared in the FTIR spectrum of both pure 4-NP and the 4-NP-templated chitosan MIP membrane, but were absent from the FTIR spectra of the chitosan membrane and the GTA-crosslinked chitosan membrane, indicating that the 4-NP molecules were immobilized in the chitosan MIP membrane. When compared with the FTIR spectra of the pure chitosan and GTA-crosslinked chitosan membranes, the existence of the 940 cm^−1^ and 1109 cm^−1^ peaks in the 4-NP-templated chitosan’s FTIR spectrum changed the appearance of chitosan’s C-O-C vibration peak at 1024 cm^−1^ [32,34], which is still visible in the recorded FTIR spectrum of the 4-NP-templated chitosan MIP membrane.

### 3.2. Negative Fiber Optic EW Absorption Spectra Confirming the Washing out of 4-NP Template Molecules from the Chitosan MIP Membrane

**Figure 3:**
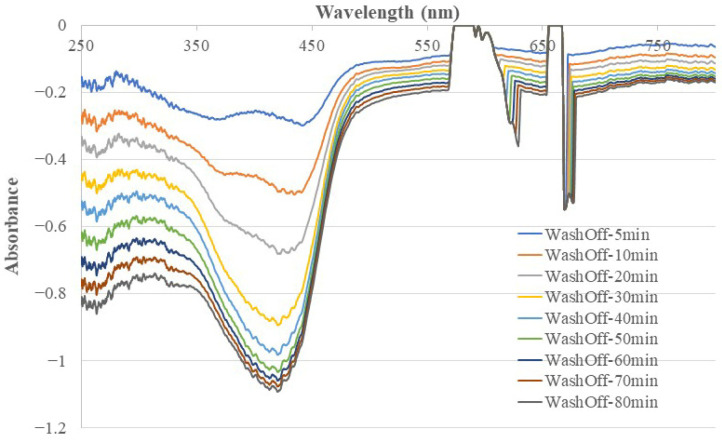
The time profile of the optical fiber EW absorption spectrum during the process of washing out 4-NP template molecules from a chitosan MIP membrane coating a BOFP. The negative absorption spectra demonstrate that all of the 4-NP molecules were washed out after 70 min of inserting the probe in DI water.

**Figure 4:**
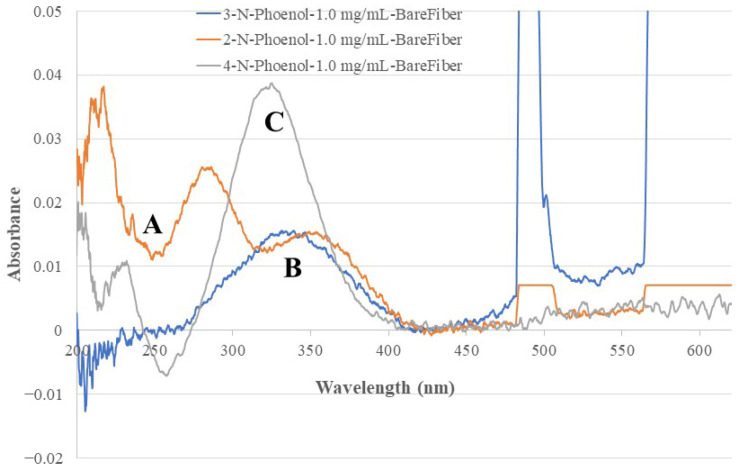
The optical fiber EW absorption spectra of 2-NP (spectrum A), 3-NP (spectrum B) and 4-NP (spectrum C) recorded with a bare-fiber BOFP (without any coating) exposed to 1.0 mg/mL standard solutions of individual NPs.

Figure 3 shows the fiber optic EW absorption spectra recorded at different times after a newly prepared chitosan-MIP-coated BOFP was first exposed to DI water. The light intensity at the moment the BOFP was inserted into DI water was recorded and used as a reference for recording these optical fiber EW absorption spectra. The negative absorption spectra with peak absorption wavelengths indicate that 4-NP was gradually washed out of the chitosan MIP membrane over time after deploying the sensor probe into DI water. Comparing the absorption spectrum recorded at 5 min after the probe was inserted into DI water and the spectra recorded at later times, it was noticed that the EW absorption spectrum at an early time had two peak absorption wavelengths, 350 nm and 440 nm. However, with more time in DI water, the absorption peak at 350 nm disappeared and the absorption peak at a longer wavelength shifted to 420 nm. The difference in the behavior of these two absorption peaks indicates that 4-NP exists in the chitosan membrane in more than one form. 4-NP in an aqueous solution absorbs light, with a peak absorption wavelength at around 330 nm, as shown in Figure 4 (spectrum C). The observed change in the absorption spectrum during the washing out process indicates that part of the 4-NP in the chitosan membrane was not in the molecularly imprinted vacancy, but distributed randomly among the chitosan chains. These 4-NP molecules can be washed out of the membrane quickly. The negative absorption peak at 420 nm was caused by slowly washing out the 4-NP molecules in the molecularly imprinted vacancy. From the spectrum intensity, it is believed that the majority of the 4-NP in the chitosan membrane was in the molecularly imprinted vacancy. Similar washing out behavior was also observed in washing out 2-NP and 3-NP from corresponding MIP membranes.

For the 4-NP-templated chitosan MIP membrane, the recorded fiber optic EW absorption spectrum stabilized after 70 min of the probe being inserted into DI water, which indicates that all of the 4-NP template molecules were washed out of the membrane.

### 3.3. GTA-Crosslinked Chitosan Membrane Extracts NPs and Improves BOFP-EW-OFCS’s Sensitivity for Analyzing NPs

In order to compare the sensitivity of the BOFP-EW-OFCS for analyzing the NPs with/without chitosan membrane SPME, a bare-fiber BOFP without any coating was first deployed to 1.0 mg/mL standard solutions of 2-NP, 3-NP, and 4-NP and a fiber optic EW absorption spectrum was recorded for each of the sample solutions. The BFOP was then coated with a GTA-crosslinked chitosan membrane (non-MIP) according to the procedure described in the Section 2. This non-MIP-chitosan-membrane-coated BOPF was then deployed to standard solutions of 2-NP (50 µg/mL), 3-NP (50 µg/mL), and 4-NP (2.0 µg/mL), and an optical fiber EW absorption spectrum was recorded for each standard solution.

**Figure 5:**
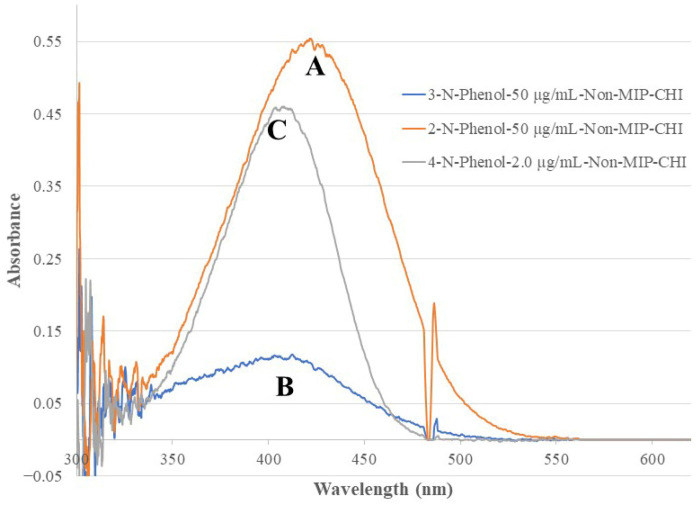
Optical fiber EW absorption spectra of 2-NP (50 µg/mL, spectrum A), 3-NP (50 µg/mL, spectrum B), and 4-NP (2.0 µg/mL, spectrum C) recorded with the same BOFP as that used in Figure 4, but coated with a non-MIP chitosan membrane.

Figure 4 shows the recorded fiber optic EW absorption spectra of the bare-fiber BOFP exposed to 1.0 mg/mL standard solutions of 2-NP, 3-NP, and 4-NP. These compounds showed absorption spectra with peak absorption wavelengths at around 350 nm and shorter wavelengths. Figure 5 shows the recorded spectra of the non-MIP-chitosan-membrane-coated BOFP exposed to NP standard solutions. Compared with the bare-fiber BOFP’s absorption spectra, two facts about the spectra recorded with the non-MIP-chitosan-membrane-coated BOFP were noticed: 1. the peak absorption wavelengths of these NPs were red-shifted to above 400 nm (410 nm to 425 nm); 2. it is much more sensitive in detecting these compounds. The peak absorption wavelength’s red-shifting by NPs extracted into the chitosan MIP membrane compared with NPs’ absorption spectra obtained with the bare-fiber BOFP was caused by a difference in the environment the NPs were exposed to. In the case of the bare-fiber BOFP test, the NPs were in an almost pH neutral aqueous solution. When extracted into the non-MIP chitosan membrane, the NPs were in a weak basic environment due to the existence of amine groups in chitosan’s side chain. The red-shifting of the peak absorption wavelength of the organic acids extracted into the chitosan membranes was also observed from our previous works [23,24,25,26,27].

The non-MIP-chitosan-membrane-coated BOFP showed a much higher sensitivity when compared with the bare-fiber BOFP for detecting the NPs, but the sensitivity improvement is different for each individual NP. A sensitivity improvement factor (SIF) was calculated by using following equation to quantitatively compare the sensitivity improvement for each NP:

(1) $$\mathrm{SIF}=\frac{{\left(\mathrm{Abs}@\lambda_{\max}\right)}_{\mathrm{non}\text{-}\mathrm{MIP}}/C_{\mathrm{NP}}}{{\left(\mathrm{Abs}@\lambda_{\max}\right)}_{\mathrm{BareFiber}}/C_{\mathrm{NP}}}$$

Table 1 lists the peak absorption wavelengths used (λ_max_) and the calculated SIF value for each individual NP. 4-NP has the highest SIF value and 3-NP has the lowest SIF value. The sensitivity improvement for a BOFP coated with a non-MIP chitosan membrane is a SPME result, as diagrammatically shown in Figure 1 (Graph B). The crosslinked chitosan membrane on the surface of a BOFP extracts and concentrates NPs into the membrane. The chitosan membrane has a basic microenvironment and the acidic property of the NPs benefits this equilibrium toward concentrating the NPs into the non-MIP chitosan membrane. The nitro group (-NO_2_) on the ortho (2) and para (4) positions improves the phenol group’s acidity much more than the nitro group on the meta (3) position, as indicated by the pKa values listed in Table 1. Therefore, the chitosan membrane extracts 2-NP and 4-NP more effectively than 3-NP. Among 2-NP and 4-NP, a steric factor affects the extraction of these compounds into the chitosan membrane. The nitro group in the orthro position is too close to the phenol group, which sterically blocks the attraction of 2-NP to the amine group in chitosan, which results in a smaller SIF value when compared with 4-NP, which does not have this steric block-out issue.

### 3.4. A 4-NP-Templated Chitosan MIP Membrane Coating on a BOFP Improves the Sensitivity of a BOFP-EW-OFCS for Analyzing 4-NP

**Figure 6:**
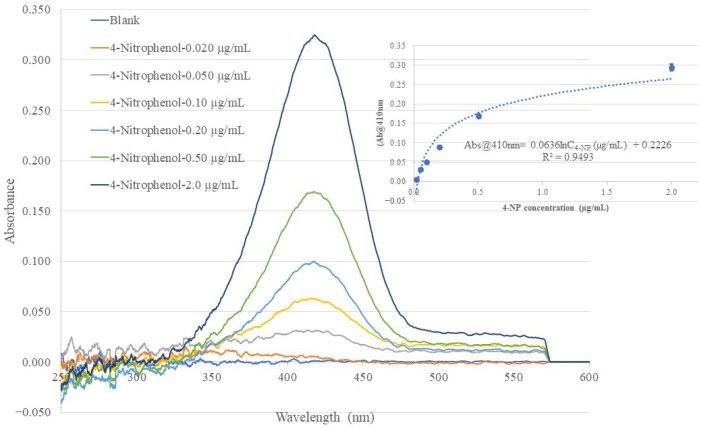
The optical fiber EW absorption spectra of 4-NP standard solutions of different concentrations recorded using the same BOFP as that used in recording the spectra in Figure 4 and Figure 5, but coated with a 4-NP-templated chitosan membrane. The inserted graph shows the relationship of the recorded spectrum’s peak wavelength (410 nm) absorbance with the 4-NP concentration in the standard solutions. Each data point presented in the inserted curve was the average response of three tests.

**Figure 7:**
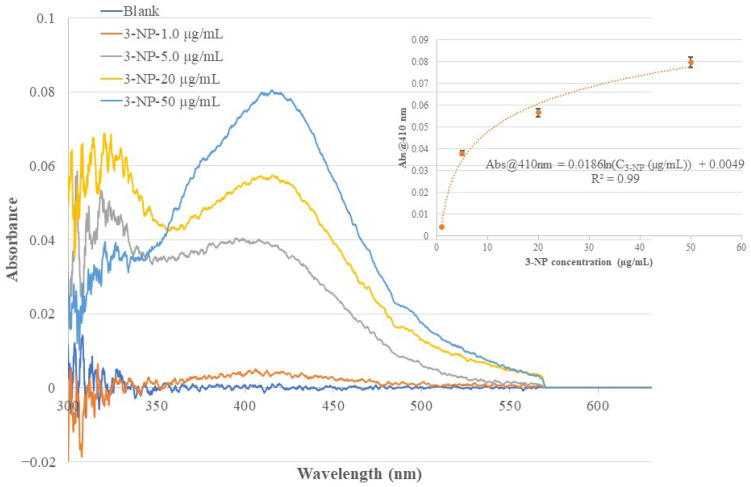
The optical fiber EW absorption spectra of 3-NP standard solutions of different concentrations recorded using the same BOFP as that used in recording the spectra in Figure 6, but coated with a 3-NP-templated chitosan membrane. The inserted graph shows the relationship of the recorded spectrum’s peak wavelength (410 nm) absorbance with the 3-NP concentration in the standard solutions. Each data point presented in the inserted curve was the average response of three tests.

**Figure 8:**
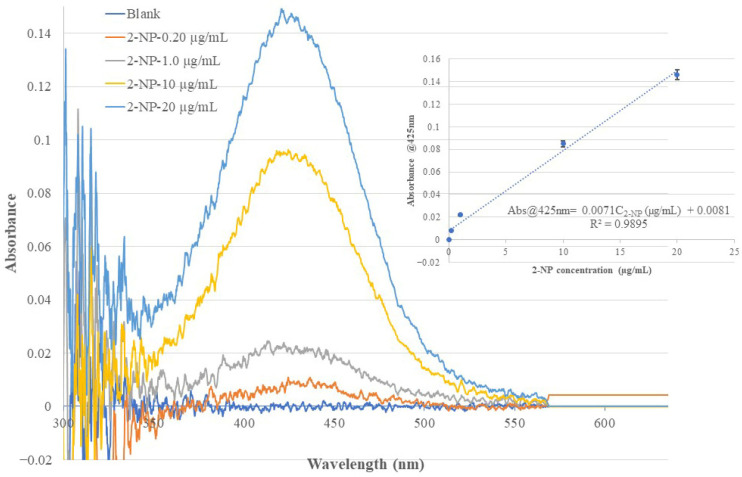
The optical fiber EW absorption spectra of 2-NP standard solutions of different concentrations recorded using the same BOFP as that used in recording spectra in Figure 6, but coated with a 2-NP-templated chitosan membrane. The inserted graph shows the relationship of the recorded spectrum’s peak wavelength (425 nm) absorbance with the 2-NP concentration in the standard solutions. Each data point presented in the inserted curve was the average response of three tests.

In this work, the same BOFP used in the bare-fiber test and non-MIP membrane test was used to coat different chitosan MIP membranes with 2-NP, 3-NP, and 4-NP as templates. The BOFP coated with different MIP membranes was analyzed for the corresponding template compound. In the test, the coated BOFP was deployed to standard solutions of the analyte of different concentrations and an EW absorption spectrum was recorded for each standard solution. Figure 6, Figure 7 and Figure 8 show the recorded absorption spectra of the chitosan-MIP-membrane-coated BOFP for analyzing 4-NP, 3-NP, and 2-NP. The inserted graph in each of these figures shows the quantitative relationship of the sensor’s absorbance value with the analyte’s concentration in the standard solutions.

In MIP-based sensor techniques, a parameter called imprinting factor (IF) was used to describe the sensitivity improvement by comparing the sensitivity of a transducer coated with an MIP membrane and the same transducer coated with a non-MIP membrane [24,26]. The IF values for the three chitosan-MIP-membrane-coated BOFPs with 2-NP, 3-NP, and 4-NP as templates were calculated using following equation:

(2) $$\mathrm{IF}=\frac{{\left(\mathrm{Abs}@\lambda_{\max}\right)}_{\mathrm{MIP}}/C_{\mathrm{NP}}}{{\left(\mathrm{Abs}@\lambda_{\max}\right)}_{\mathrm{non}\text{-}\mathrm{MIP}}/C_{\mathrm{NP}}}$$

The calculated IF values for the three NP-templated chitosan MIP membranes are listed in Table 1. It was noticed that the 4-NP-templated chitosan MIP membrane coating on the BOFP improved the sensitivity of the MIP-SPME-EW-OFCS toward 4-NP with a 2.3 IF value. However, the BOFP coated with a 2-NP- or a 3-NP-templated MIP membrane did not improve the sensor’s sensitivity (IF value < 1) in analyzing 2-NP and 3-NP, respectively. It was believed that 3-NP is a very weak acid. Its interaction with amine group on chitosan’s side chain is not strong enough to imprint the molecule with an amine group in the formed polymer. In the case of 2-NP, it was believed that the steric factor prevented the effective bonding of its phenol group with amine in chitosan’s side chain and thus it could not form an MIP membrane.

In considering the acidity, steric factor, and IF value, it was concluded that a BOFP coated with a 4-NP-templated chitosan MIP membrane can achieve a high sensitivity improvement (SIF = 1.4 × 10^4^ when compared with a bare-fiber BOFP) for analyzing 4-NP, but BOFP coated with a 2-NP-templated chitosan MIP membrane or a 3-NP-templated chitosan MIP membrane does not display much sensitivity improvement in analyzing the respective compound. Therefore, this work was focused on the development of an MIP-SPME-EW-OFCS for analyzing 4-NP.

### 3.5. Reversibility, Response Time, and Stability of the MIP-SPME-EW-OFCS for Monitoring 4-NP

**Figure 9:**
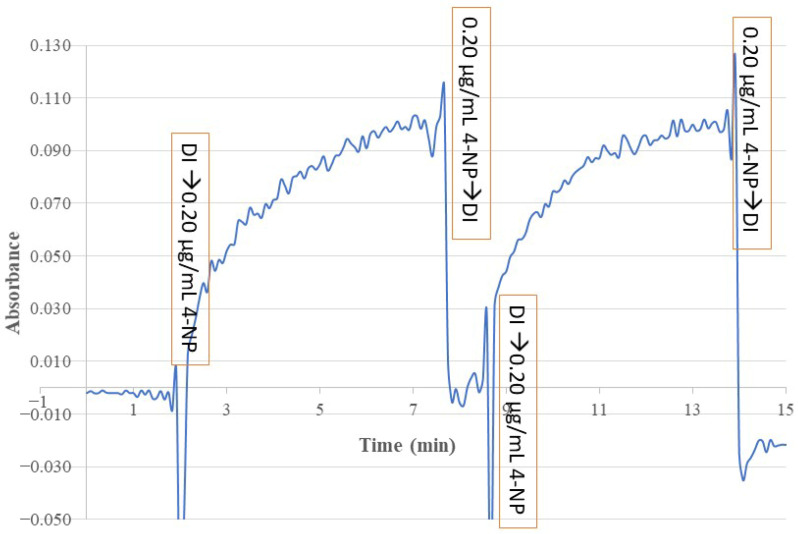
The time response of a 4-NP-templated chitosan-MIP-membrane-coated BOFP alternatively exposed to DI water and a 0.20 µg/mL 4-NP standard solution. The spikes in the time profile were caused by changing of the sample solution between DI water and the 4-NP standard solution.

The reversibility and time response of this sensor for monitoring 4-NP in a standard solution was investigated by alternately exposing the sensing probe to DI water and a 0.20 µg/mL 4-NP standard solution and continuously monitoring the BOFP’s EW absorbance at 410 nm over time. Figure 9 shows the test result. This test result demonstrates the reversible response nature of this sensor. A reversible response is necessary for using a sensor to continuously monitor a sample in a process line, such as in environmental remediation to remove a water contaminate from a polluted waterbody. This sensor’s reversible response originates from the sensor’s response mechanism, MIP-membrane SPME, which is a reversible process and does not involve any chemical reactions.

The response time of the sensor is defined as the time needed for the sensing signal to reach 90% of its full response after deploying the sensor probe to a sample solution. From the time response test result shown in Figure 9, the response time of this sensor was calculated to be 5 min.

The transducer of this 4-NP sensor is a BOFP coated with a GTA-crosslinked chitosan membrane with a special pore structure (4-NP imprinted MIP). The GTA-crosslinked chitosan is a stable polymer even with long-term exposure to water or a weak basic solution [35,36]. However, this polymer can be dissolved in an acidic solution. This sensor is expected to be stable in long-term use as long as the sensing probe is not exposed to a strong acidic solution. During this research work, a single 4-NP-templated chitosan-MIP-coated BOFP was used to collect all the experimental data, involving testing the sensor’s response to 4-NP and interference species and sample analysis. During the three months’ experiments, the sensor demonstrated a stable response to 4-NP, which verified the long-term stability prediction.

### 3.6. Calibration Curve and Detection Limit

**Figure 10:**
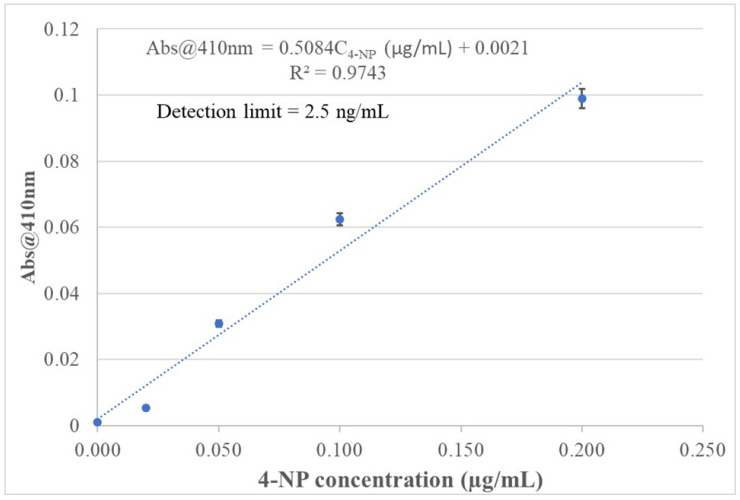
A linear calibration curve describes the relationship of the 4-NP sensor’s absorbance at 410 nm, with the 4-NP concentration in standard solutions in a narrow concentration range from blank to 0.20 µg/mL. Each data point presented in the inserted curve was the average response of three tests.

The data from the spectral response of the 4-NP-templated chitosan-MIP-membrane-coated BOFP to 4-NP in standard solutions were used to establish calibration curves for quantitatively analyzing 4-NP in an aqueous sample solution. The inserted graph in Figure 7 shows that the sensor’s absorbance signal at 410 nm has a logarithmic relationship with the 4-NP concentration in standard solutions. This relationship agrees with the fact that the chitosan MIP membrane is a porous material. The distribution equilibrium of 4-NP between the sample solution and the chitosan MIP membrane follows the Langmuir isotherm, which describes the equilibrium of adsorbing a compound onto the pore surface of a porous material to form a single molecular layer of the adsorbed compound. In this work, 4-NP concentration in the tested standard solutions is in the µg/mL range and a single layer of adsorbed 4-NP on the chitosan MIP pore surface was expected. In a narrow 4-NP concentration range from blank to 0.20 µg/mL, the sensor’s absorbance value at the peak absorption wavelength can be approximated to have a linear relationship with the 4-NP concentration in standard solutions, as shown in Figure 10, with a good correlation coefficient (R^2^ = 0.974).

The detection limit of the sensor is defined as the 4-NP concentration in a sample solution which causes a sensing signal that is three times the standard deviation (STDV) of a blank signal above a blank sample’s sensing signal. The blank sample signal’s STDV was calculated from the time response test result in Figure 9. The smallest STDV from 10 consecutive blank samples’ sensing signal was calculated to be 0.00042. The detection limit calculated from the STDV value and slope of the linear calibration shown in Figure 10 is 2.5 ng/mL (0.018 µM). Although optical fiber EW absorption spectrometry has limited sensitivity in detecting a light-absorbing compound in the fiber’s cladding layer due to a limited interaction pass-length (µm range) [28,29,30], the sensor of this work achieved a detection limit which is lower than that of traditional UV/Vis absorption spectrometry coupled with HPLC [14,17,37]. This HPLC separation with UV/Vis spectrometric detection methods for analyzing 4-NP has reported a detection limit in the range of 105 ng/mL [37] to 150 ng/mL [17]. The high concentration factor (1.4 × 10^4^) of extracting 4-NP from a sample solution into the chitosan MIP membrane improved the sensor’s sensitivity and helped to achieve the low detection limit.

### 3.7. Comparison of the Responses of the MIP-SPME-EW-OFCS to 4-NP and Its Isomers, Derivatives, and Other Compounds

**Figure 11:**
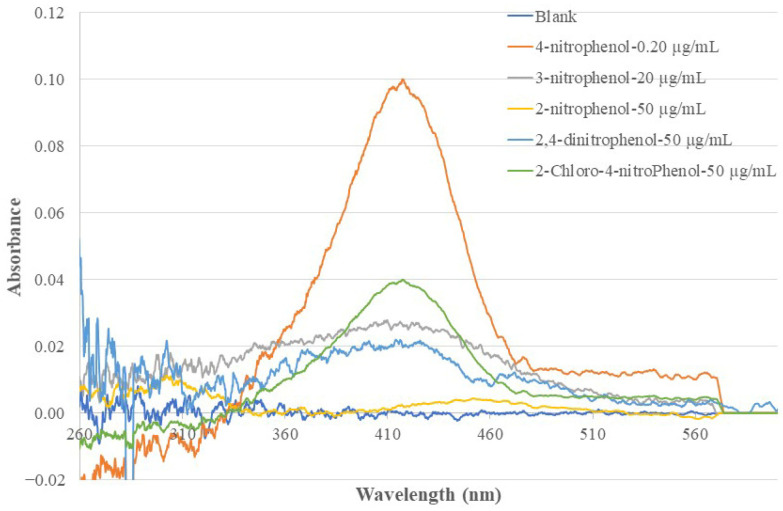
Optical fiber EW absorption spectra of a 4-NP-templated chitosan-MIP-coated BOFP exposed to standard solutions of 4-NP, its positional isomers (2-NP and 3-NP) and its derivatives (2,4-di-NP and 2-chloro-4NP).

The sensor’s response to 4-NP, its isomers (2-NP and 3-NP), and two randomly selected derivatives (2,4-di-NP and 2-chloro-4-NP) was investigated. In such a test, the sensor probe was deployed to a standard solution of the selected compound and the resulting EW absorption spectrum was recorded. Figure 11 shows the recorded absorption spectra. A selectivity factor (SF), which describes the ratio of the sensor’s absorbance at 410 nm caused by 4-NP of unit concentration to the absorbance at the same wavelength caused by an interference species of unit concentration, is calculated using the following equation:

(3) $$\mathrm{SF}=\frac{{\left(\mathrm{Abs}@410\mathrm{nm}\right)}_{4\text{-}\mathrm{NP}}/C_{4\text{-}\mathrm{NP}}}{{\left(\mathrm{Abs}@410\mathrm{nm}\right)}_{\mathrm{InterfSpec}}{/C}_{\mathrm{InterfSpec}}}$$

The calculated SF values for the tested compounds are listed in Table 2. These SF values indicate that this sensor’s response to 4-NP is >100 times more sensitive than its response to the tested isomers and derivatives. This ensures that the existence of other NP isomers and derivatives will not cause interference to the sensor’s function of analyzing 4-NP if 4-NP, its isomers, and its derivatives co-exist in a sample solution in the same concentration magnitude. The SF value for a 4-NP sensor using a non-MIP-membrane-coated BOFP was also calculated using the data shown in Figure 4 and the calculated SF values are listed in Table 2. A selectivity improvement factor (SLIF) was calculated by dividing the MIP-based sensor’s SF value by the non-MIP-based sensor’s SF value. The calculated results listed in Table 2 indicate that, compared with the non-MIP-membrane-based 4-NP sensor, the MIP-based 4-NP sensor’s selectivity toward its isomers increased by 18–20 times. The chitosan-MIP-membrane-based sensor has an IF value of 2.3 when compared with the non-MIP-chitosan-membrane-based sensor for analyzing 4-NP. It is reasonable that this IF contributed to the SLIF values. However, the SLIF values are much larger than this IF value and there could be other factors that contributed to the improved SLIF values. One considerable factor that contributed to the large SLIF value is that in a 4-NP-templated chitosan MIP membrane, the vacancy inside the membrane was imprinted for 4-NP. When compared with a non-MIP-based membrane, there are far fewer sites available for 2-NP or 3-NP to compete to bond with in this MIP membrane and less chance the 2-NP and 3-NP being extracted into the MIP membrane.

Table 2 also lists the calculated SF values using data from a published electrochemical 4-NP sensor with single-wall carbon nanotube modified carbon glass electrode as the transducer [13]. The sensor in this work demonstrated much better selectivity in discriminating 4-NP from its positional isomers when compared with the electrochemical sensor technique.

The sensor’s responses to several other substances were also investigated. The existence of the following tested compounds in a sample solution in the concentration indicated in the bracket following the compound’s name was found not to interfere with the sensor’s function of analyzing 4-NP in an aqueous solution sample: Na_2_CO_3_ (50 mM), urea (1.0 mg/mL), cane sugar (1.0 mg/mL), KCl (1.0 mg/mL), citric acid (100 µg/mL), and acetylsalicylic acid (50 µg/mL).

### 3.8. Analyzing 4-NP in a Standard Addition Sample

A bottled water drink product purchased from a local grocery store was used as a sample. A volume of 1.0 mL of a 2.0 µg/mL 4-NP standard solution was added to 19.0 mL of bottled water to make a test standard addition sample with a 4-NP concentration of 0.10 µg/mL. This sample was analyzed with the sensor in this work using a calibration curve established with the 4-NP standard solutions for quantitation. The analytical result is listed in Table 3, with the recovery rate ranging from 93% to 101%, indicating the feasibility of using the developed sensor for analyzing 4-NP in water samples.

## 4. Conclusions

An OFCS for selectively analyzing 4-NP in aqueous solution samples was developed by integrating MIP-SPME with BOFP EW absorption spectrometry. The chitosan MIP membrane with 4-NP as a template fabricated on the surface of a BOFP selectively extracts 4-NP and concentrates the analyte by 1.4 × 10^4^ times into the membrane for fiber optic EW absorption spectrometric detection. Although chitosan MIP membranes using 2-NP or 3-NP as a template were also fabricated and coated on the surface of the same BOFP, these MIP membranes did not demonstrate specific improvement in extracting the respective template molecules into the membranes. It was believed that the template molecule’s acidity and molecular structure played critical roles in deciding a MIP membrane’s capability of concentrating the analyte into the membrane for detection. The sensor of this work, for analyzing 4-NP, achieved a detection limit of 2.5 ng/mL, which is lower than the detection limit of the reported traditional UV/Vis spectrometric method for analyzing this compound in an aqueous sample solution. The sensor also demonstrated high selectivity. The existence of 4-NP’s isomers and derivatives does not cause interference to the sensor’s function of analyzing 4-NP in an aqueous solution sample. The results from analyzing 4-NP in a standard addition sample illustrate the feasibility of using this sensor for analyzing 4-NP in water samples.

## Figures and Tables

**Table 1:** SIF values comparing a chitosan-membrane (MIP or non-MIP)-coated BOPF with a bare-fiber BOFP for an EW-OFCS analyzing NPs and IF values comparing a chitosan-MIP-membrane-coated BOFP with a non-MIP-chitosan-membrane-coated BOFP for analyzing NPs.

Analyte	λ_max_	SIF (Non-MIP/BareFiber)	pKa	IF of MIP Membrane	SIF (MIP/BareFiber)
2-NP	350 nm (bare fiber) 425 nm (chitosan membranes)	7.4 × 10^2^	7.2	0.66	4.9 × 10^2^
3-NP	335 nm (bare fiber) 410 nm (chitosan membranes)	1.5 × 10^2^	8.4	0.71	1.2 × 10^2^
4-NP	325 nm (bare fiber) 410 nm (chitosan membranes)	6.0 × 10^3^	7.1	2.3	1.4 × 10^4^

**Table 2:** The SF and SLIF values comparing the sensitivity of a 4-NP-templated chitosan-MIP-membrane-coated BOFP to 4-NP and its isomers and selected derivatives.

Compound	SF-MIP	SF-Non-MIP	SLIF	SF-Electrochemical Sensor
4-NP	1	1	1	1
2-NP	1922	97	20	177
3-NP	372	21	18	190
2-Cl-4-NP	104	NA *	NA	NA
2,4-di-NP	140	NA	NA	146

*: data are not available to calculate the value.

**Table 3:** The analytical results of using this sensor for analyzing 4-NP in a standard addition sample with a bottled water drink product as sample matrix.

Test Number	4-NP Concentration Added to Test Sample (µg/mL)	4-NP Concentration Measured with This Sensor (µg/mL)	Recovery Rate
Test 1	0.10	0.093	93%
Test 2	0.10	0.101	101%
Test 3	0.10	0.101	101%
Average	0.10	0.098	98%
STDV/RSD%		0.0046/4.7%	

## Data Availability

The original contributions presented in this study are included in the article. Further inquiries can be directed to the corresponding author.
